# Central-European sunshine hours, relationship with the Atlantic Multidecadal Oscillation, and forecast

**DOI:** 10.1038/s41598-024-73506-5

**Published:** 2024-10-24

**Authors:** Horst-Joachim Lüdecke, Gisela Müller-Plath, Sebastian Lüning

**Affiliations:** 1 University of Applied Sciences htw, Saarbrücken, Germany; 2 Technische Universität Berlin, Berlin, Germany; 3 Institute for Hydrography, Geoecology and Climate Sciences, Hauptstraße 47, Ägeri, 6315 Switzerland

**Keywords:** Sunshine hours (SSH), Atlantic Multidecadal Oscillation (AMO), Prediction of dimming, Fourier transform analysis, Non-linear optimization, Climate sciences, Environmental sciences

## Abstract

Sunshine hours (SSH) is an important meteorological parameter, loosely linked to temperature and precipitation, and highly relevant for various sectors such as agriculture or solar energy. Previous studies have already identified a correlation of European SSH with the thermal state of the North Atlantic. This paper investigates this relationship further by studying annual and monthly SSH of seven long-term Central European SSH series and comparing them to the Atlantic Multidecadal Oscillation (AMO) using Fourier Transformation, Monte Carlo simulation and non-linear optimization. The Fourier spectra of our annual SSH series have their strongest and highly significant peaks in the known AMO period of ~ 50 to ~ 80 years, supporting the hypothesis that European SSH and the AMO are linked. The optimized sinusoids of the seven SSH and the AMO series with these periods show substantial correlations with the corresponding data (*r* = 0.42–0.55 for SSH and 0.71 for the AMO). Extrapolating the sinusoids, we project a gradual decline in SSH across Central Europe by 9–16% from its current maximum over the next three decades, particularly pronounced in northern regions.

**Supplementary Information:**

The online version contains supplementary material available at 10.1038/s41598-024-73506-5.

The sunshine duration varies from year to year, but also from decade to decade in the form of long-term trends and oscillations. Sunshine hours (SSH) as annual, seasonal or monthly totals is a standard parameter that has been measured in weather stations for many decades. Accurate information on SSH is of great importance for several applied sectors such as agriculture, tourism, solar energy, and household/transport energy consumption (fewer SSH per day require more hours of artificial lighting). Understanding trends in SSH enables early planning of mitigation measures. For example, farmers may invest in technology that compensates for some of the variations in SSH, solar energy producers may better forecast their yields and hence commercial revenues^1^, and in cooler regions, summer holiday destinations may offer additional indoor attractions to bridge extended periods of cloud cover.

The distribution of SSH in different regions of the world and its relationship with air temperature, North Pacific and North Atlantic sea surface temperatures (SST), North Atlantic thermohaline circulation, precipitation, aerosols and biosphere growth have been studied for some time. For example, temporal trends in SSH have been reported over the Iberian Peninsula from 1931 to 2004^2^, over Switzerland since the late 19th century^3^, over Europe from 1939 to 2012 and from 1961 to 2010^4,5^, over the western part of Europe from 1938 to 2004^6^, over the USA from 1996 to 2019^7^, over Japan from 1960 to 2015^8^, and over Northeast India from 1965 to 2000^9^. Analyzing the relationship between SSH and air temperature for the Polish city of Krakow from 1884 to 2010^10^ and for 312 stations in Europe^5^ has revealed a moderate to high correlation between daily sunshine duration and both daily maximum temperature and daily temperature range, but only during summer and fall. The relationship between SSH over the Northern Hemisphere continents and SST variations in the North Pacific and North Atlantic has been reported^11^, and also between SSH over Europe and the thermohaline circulation in the North Atlantic, where the latter accounted for one third of the variability in the first^12,13^. Evidence has also been found for aerosol-induced changes in SSH^14^. Although SSH correlates with air temperature or precipitation in some seasons or regions^5,10^, the relationship is not straightforward: A sunny winter day can be excessively cold, and an overcast day may stay dry. SSH is therefore an important climate parameter in its own right and deserves more attention than it has received in the past.

In this paper, we investigate a possible correlation of SSH data with an important Atlantic mode of variability, the Atlantic Multidecadal Oscillation (AMO). The AMO is based upon the average anomalies of SST^15,16^. It typically develops as a succession of negative and positive phases of the SST in the North Atlantic, each lasting roughly three to four decades^17^. For the first time, we use Fourier Transformation, Monte Carlo simulation and non-linear optimization to determine the degree to which the SSH in Central Europe is coupled with the sinusoidal AMO over a long period of time (1856–2022) and apply the extracted sine to predict future SSH. Since the AMO cycle has a period of about 70 years and Fourier analyses requires at least one and a half times the period of interest to be reliable, we looked for SSH time series with lengths of at least 120 years. Further criteria for selection of SSH series were that they should come from single stations, have only very few data gaps (tolerated only in the year 1945 in which gaps might be unavoidable, and only for less than six months), and be openly accessible. We used the following seven stations that met these requirements, five from Central Europe proper and two in the immediate vicinity: Copenhagen/Denmark, 1876–2020; Potsdam/Germany, 1893–2022; De Bilt/Netherlands, 1901–2022; Krakow/Poland, 1884–2023; Vienna/Austria, 1881–2022; Zugspitze/Germany, 1901–2022; Trieste/Italy, 1886–2006. There were no data gaps for Trieste, Vienna, Copenhagen and Krakow. The months May to August 1945 were missing for Zugspitze, April 1945 for De Bilt and April and May 1945 for Potsdam. Other stations meeting the criteria were not available.

Linking SSH to well-established oceanic oscillations is then used to better forecast the future long-term development of SSH in Central Europe. The annual SSH of the studied time series and the AMO are shown in Fig. 1.

**Fig. 1:**
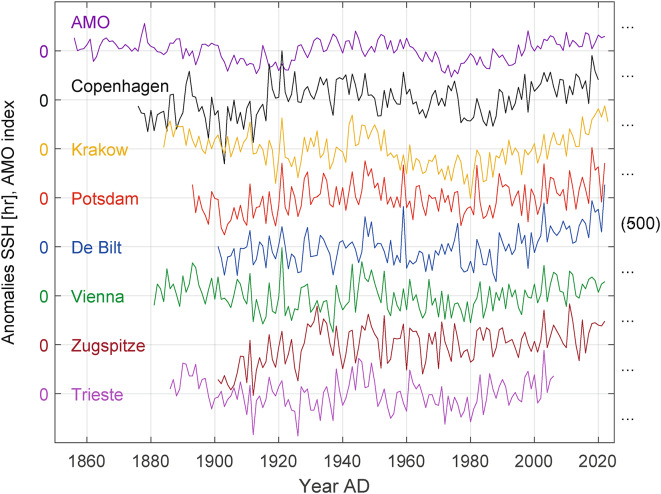
Time series of total annual SSH plotted as anomalies and the AMO index (anomaly by definition) with y - grid lines equally spaced at 500 hr intervals. The AMO index has been multiplied by 50 for illustration purposes*.*

All seven long-term SSHs and the AMO resemble a sinusoid even with the naked eye. Therefore, we applied the Fast Fourier Transform with zero padding^18^ to both the annual and monthly SSH and AMO data, except for Krakow, where only annual data were available. Significance lines with p-values of *p* = 0.001, *p* = 0.01 and *p* = 0.05 were computed with Monte Carlo simulations (MC) to confirm the statistical significance of the Fourier results (see the Methods section for details).

## Results

**Fig. 2:**
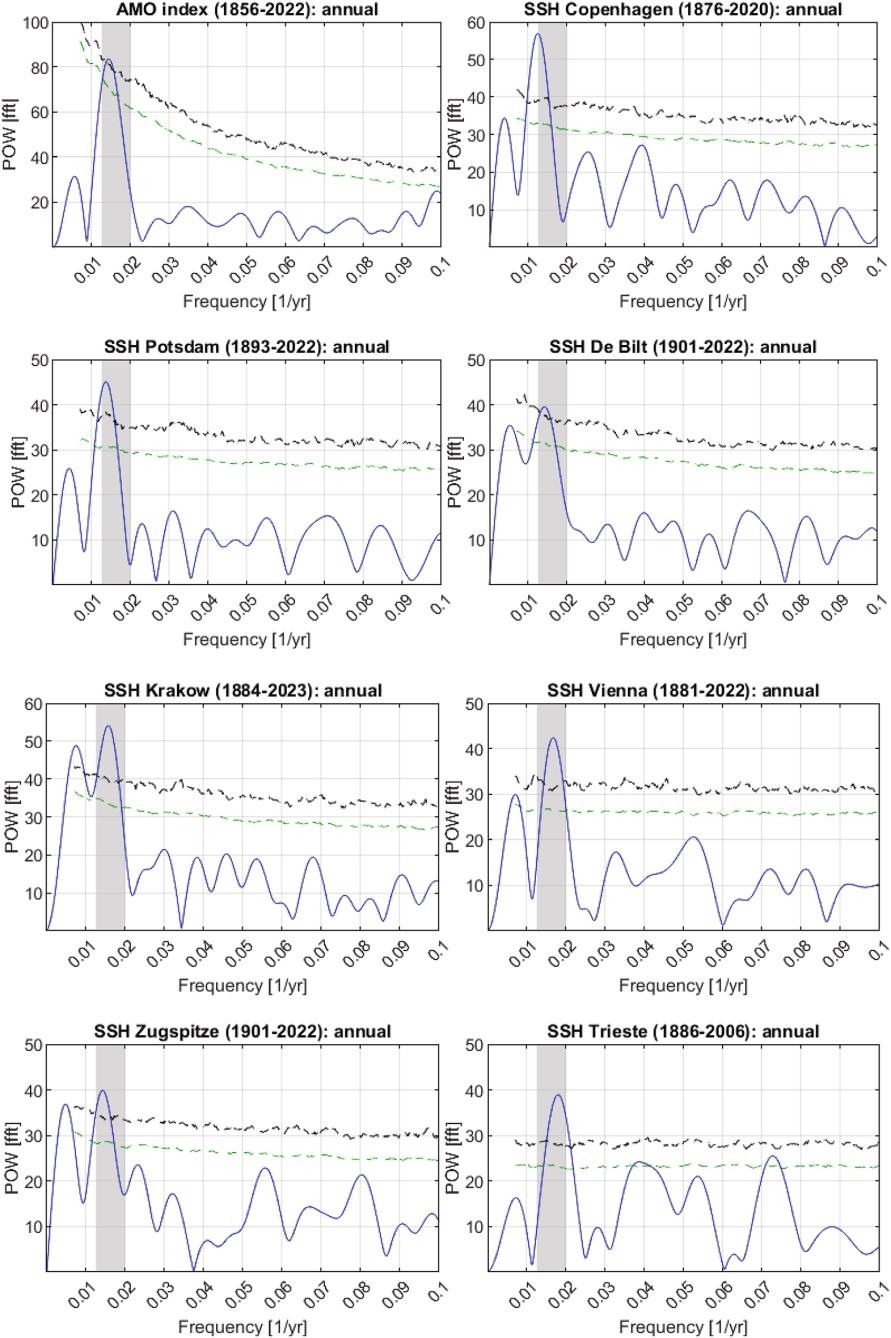
FFT spectra of the annual AMO and the total annual SSH from Copenhagen, Potsdam, De Bilt, Krakow, Vienna, Zugspitze and Trieste. The AMO sector of periods ~ 50 to ~ 80 years (AMO-CYC, frequencies between ~ 0.0125 to ~ 0.02 yr^−1^) is marked by the grey shaded area for clarity. POW [fft]: spectral power from FFT; black dashed lines: significance lines for p = 0.001; green dashed lines: significance lines for p = 0.01*.*

The Fast Fourier Transforms (FFT) revealed that the periods of the main cycle of the annual AMO index and the total annual SSH for Copenhagen, Potsdam, De Bilt, Krakow, Vienna, Zugspitze and Trieste are all between ~ 50 and ~ 80 years, supporting the idea that the AMO and the SSH may be linked (see Fig. 2).

Table 1 shows general parameters of the total annual SSH and the annual AMO index together with results of the FFT analysis. There is a striking north-south decrease for the SSH periods, with the exception of Zugspitze. Between 10 years and ~ 50 - ~80 years (AMO-CYC), there are no other periods of similar significance.

**Table 1:** Parameters and results of the Fourier analysis for the total annual SSH and the annual AMO index. AMO-CYC period: period in the Fourier spectrum between ~ 50 and ~ 80 year or frequencies between ~ 0.0125 and ~ 0.2 yr^−1^; AMO-CYC p-value: p-value of AMO-CYC periods according to Fourier spectrum significance lines; Mean: SSH mean over the total time of the SSH series; SD: standard deviation of the SSH and the AMO; α: generalized Hurst exponent from the Detrended Fluctuation Analysis DFA used to calculate the significance lines of the Fourier spectra (for α and DFA see details under statistical Processing in the Methods section*).*

SSH total annual	Observation length [yr]	North, East, height above sea level [m]	AMO-CYC period [yr]	AMO-CYC *p*-value	Mean [hr]	SD [hr]	DFA α
AMO index	167	–	70	< 0.001	0 by def.	0.182	1.07
SSH Copenh.	145	55.7 N, 12.6 E, ~0	80	< 0.001	1604	184.9	0.6
SSH Potsdam	130	52.4 N, 13.1 E, ~ 32	73	< 0.001	1733	173.0	0.6
SSH De Bilt	122	52.1 N, 5.18 E, ~0	71	< 0.001	1575	179.3	0.63
SSH Krakow	140	50.1 N, 19.9 E, ~ 300	64	< 0.001	1576	177.4	–
SSH Vienna	142	48.2 N, 16.4 E, ~ 150	60	< 0.001	1993	157.5	0.52
SSH Zugspitze	122	47.4 N, 11 E, ~ 2960	70	< 0.001	1826	195.3	0.59
SSH Trieste	121	45.6 N, 13.8 E, ~2	56	< 0.001	2183	159.2	0.5

For the sake of simplicity, most of the following descriptions will be called “SSH” only, but refer also to AMO. Fitting the annual SSH with optimal AMO-CYC sinusoids requires two additional parameters, the phase and amplitude of the sinusoid, in addition to the known period from the Fourier analysis. We determined the phase and amplitude using the nonlinear optimization method of Nelder and Mead^19^ (see the Methods section for details).

The resulting optimal sinusoids of the SSH series are by definition a continuous long-term cyclical trend. However, there could exist additional linear trends in the SSH series, for example due to increasing atmospheric CO_2_ or other non-cyclical forcings. To determine such linear trends in the SSH separately from the sinusoidal trend, we subtracted the optimal sinusoid from the SSH. This resulted in an SSH detrended from its sinusoid, which we will refer to hereafter as SSH* and AMO*.

**Fig. 3:**
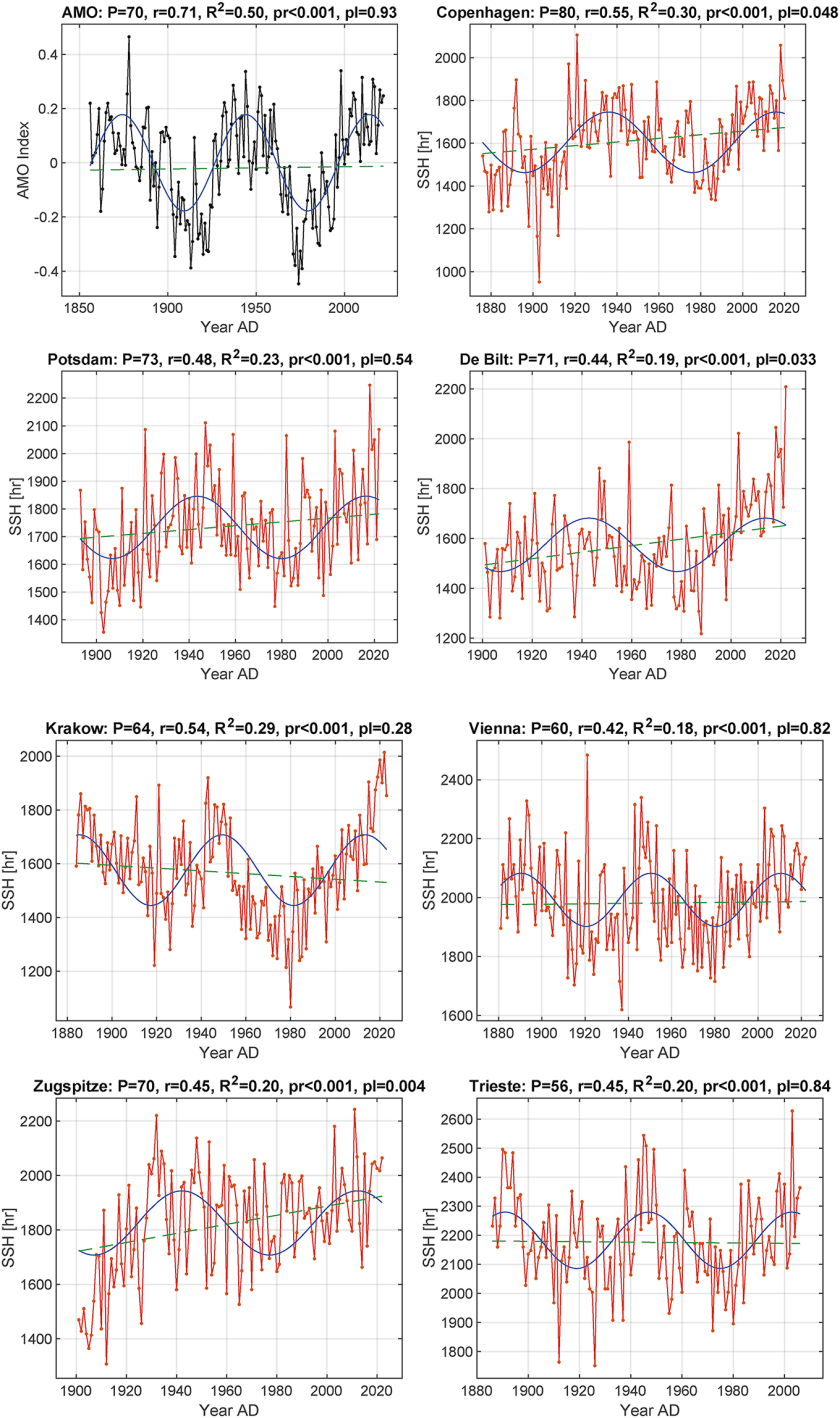
Time series of the annual AMO index (black) and the annual SSH (red) for Copenhagen, Potsdam, De Bilt, Krakow, Vienna, Zugspitze and Trieste, with their corresponding optimal sinusoids (blue) and linear regression lines for SSH* and AMO* (dashed green). P: period of the AMO-CYC in years; r: Pearson correlation between SSH and sinusoid; R^2^ = r^2^: goodness of fit, the proportion of variation in the response variable captured by the regression^20^; pr: p-value of Pearson’s r between SSH and sinusoid; pl: p-value of the slope a of the linear regression line y = a · year + b of SSH* and AMO*.

Figure 3 shows the annual time series of the AMO and the SSH for Copenhagen, Potsdam, De Bilt, Krakow, Vienna, Zugspitze, and Trieste with linear regression lines for SSH* and AMO* and their corresponding optimal sinusoids. Table 2 shows the Pearson’s correlation coefficients r of the optimal sinusoidal fit of SSH and AMO, the R^2^ = r^2^ as the quality of the fit, the statistical p-value of r, the slope of the linear regression line for SSH* and AMO*, the p-value for the slope of the regression line, and the predicted total decrease in annual SSH over the next few decades. The time interval of the decrease was fixed as ranging from the last sine maximum in the past to the next minimum in the future, which is half of the SSH period (Table 1, column 4). Note that at half of this time, the decrease of the sine curve is strongest.

**Table 2:** Results of the sinusoidal fit of SSH and AMO. r: Pearson correlation between SSH and sinusoid; R^2^ = r^2^: quality of the fit; pr: p-value of r; a: slope of the SSH*and AMO* regression line y = a · year + b; pl: p-value of the slope a; s: standard deviation of the SSH*, AMO*; dim: predicted total decrease in annual SSH during the period in brackets; dim [%]: predicted total decrease in % of the sinusoidal maximum during the period in brackets*.*

SSH, AMO annual	*r*	*R*^2^ = *r*^2^	*p*-value pr	slope a	*p*-value pl	s	dim [hr] (period), dim [%]
AMO	0.71	0.5	< 0.001	0.00008	0.93	0.128	–
SSH Copenh.	0.55	0.3	< 0.001	0.847	0.05	154.4	-283 (2016–2056), 16
SSH Potsdam	0.48	0.23	< 0.001	0.689	0.54	152.2	-225 (2017–2054,) 12
SSH De Bilt	0.44	0.19	< 0.001	1.305	0.03	160.9	-214 (2014–2049), 13
SSH Krakow	0.54	0.29	< 0.001	-0.517	0.28	149.5	-262 (2014–2046), 15
SSH Vienna	0.42	0.18	< 0.001	0.0741	0.82	142.9	-180 (2011–2042), 9
SSH Zugspitze	0.45	0.2	< 0.001	1.667	0.004	174.8	-235 (2013–2048), 12
SSH Trieste	0.45	0.2	< 0.001	-0.072	0.84	142.5	-194 (2003–2031), 9

All Pearson correlation coefficients r between the annual SSH and its associated sinusoids are statistically highly significant (Table 2, column 4). However, the portion of explained variance R^2^ = r^2^ is only between 0.18 and 0.3 for the SSH and 0.5 for the AMO (Table 2, column 3). This suggests that other forcings are additionally responsible for the high variability of the SSH and AMO. However, forcings that could explain the missing 70–82% of the SSH variability and 50% of the AMO variability are not yet known. A linear increase does not (or only marginally) account for it: The p-values of the linear regression lines for SSH*, AMO* show that only the slope of Copenhagen, De Bilt and Zugspitze are significant below the threshold of *p* = 0.05, and none below the threshold of *p* = 0.001.

**Fig. 4:**
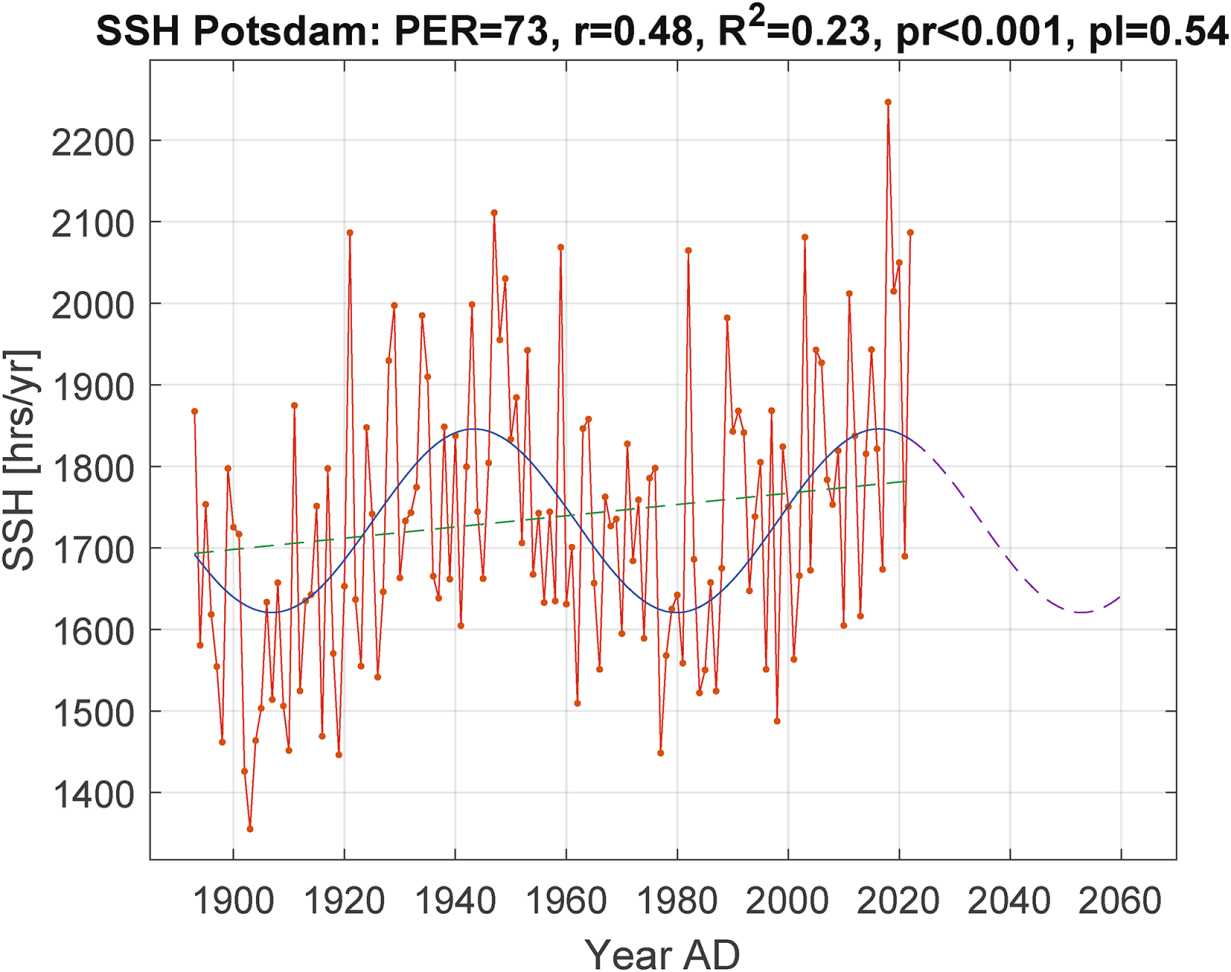
SSH Potsdam from Fig. 3 supplemented with a dimming forecast for 2017 to 2054 as the dashed part of the sinusoidal line. Since the slope of the regression line of SSH* was not statistically significant (p = 0.54, see Table 2), it was not considered for computing the forecast*.*

The predicted values of total SSH from the sinusoids show a dimming over the next 30 years or so. The southern station Vienna will be darker by a total of 180 annual SSH hours between 2011 and 2042 and Trieste by a total of 194 h between 2003 and 2031, while Northern Europe, here with Copenhagen as the northernmost station, will dim by a total of 283 h between 2016 and 2056. The total dimming is stronger in the northern countries and weaker in the southern countries, while conversely the SSH means (Table 1, column 6) are lower in the north than in the south. Figure 4 shows the sine wave forecast for the period up to 2054 for the SSH Potsdam.

**Fig. 5:**
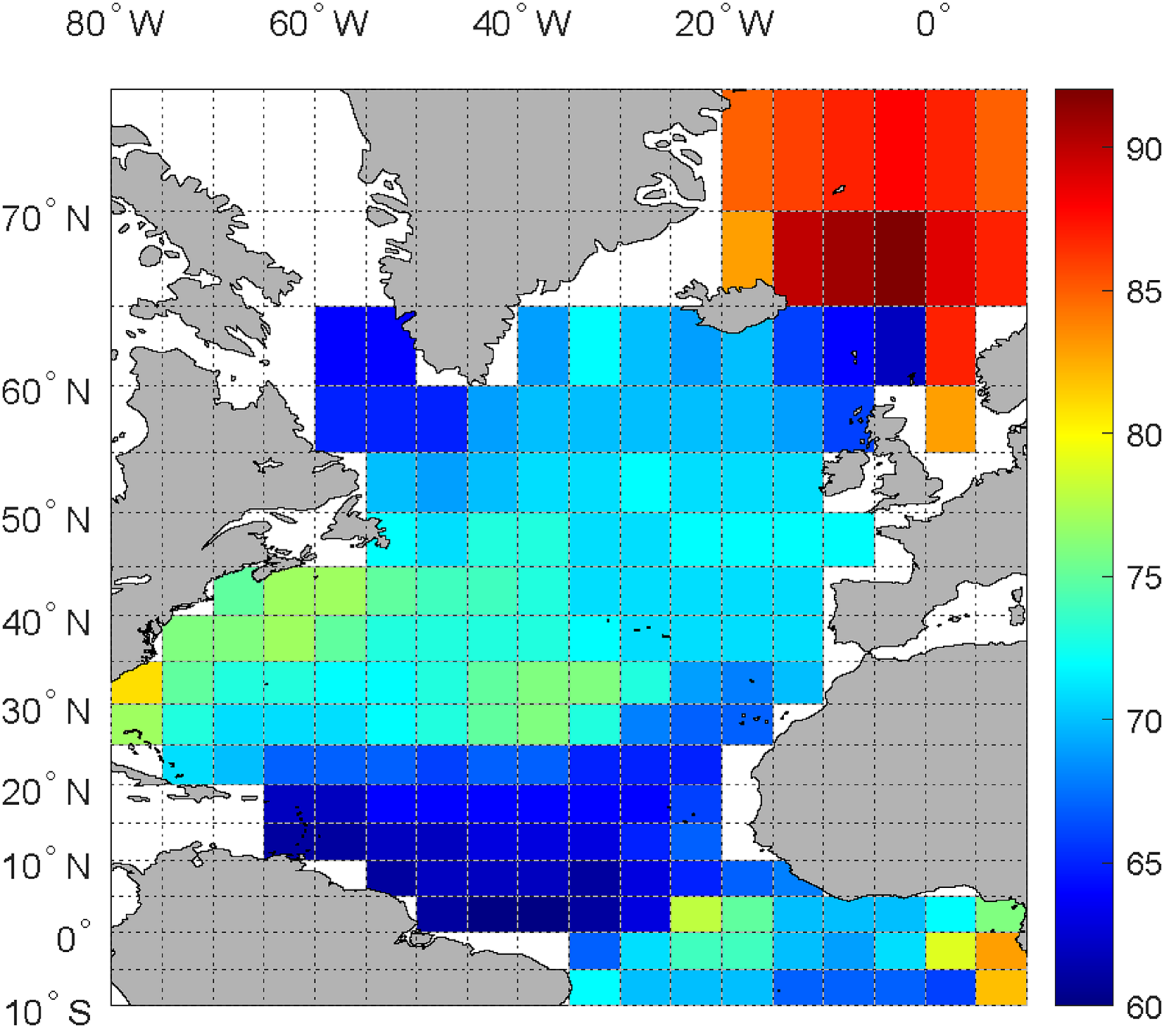
Geographical distribution of the AMO cycle periods in the 5° N x 5° E grid cells of the Atlantic region (10° S − 80° N, 10° E − 80° W). The numbers to the right of the color bar indicate the AMO periods in years computed with FFT. The numerical values of the periods are given in Table S2.

Table 1, column 4 shows annual SSH cycles with periods ranging from 56 to 80 years, while the annual AMO (1856–2022) over the North Atlantic has a cycle of 70 years. To better understand the reason for the differences between the SSH and the AMO, we additionally applied Fourier analyses to the gridded AMO data with cells of 5° N x 5° E available from 1854 to 2023^21^. As a result, we found that in the interval between ~ 50 and ~ 100 years, the periods with the greatest FFT power ranged from 60 to 92 years for different cells. Figure 5 shows the geographical distribution of these AMO periods for the 5° N x 5° E cells of the Atlantic region from 10° S to 75° N, 10° E to 80° W. The already mentioned north-south decrease of the SSH periods (Table 1, column 4) corresponds to a similar north-south decrease of the AMO periods of the eastern North Atlantic cells. (Please note that the geographical differences in periods apply only to the time since 1854 or later and do certainly not persist over centuries, otherwise the AMO would have desynchronized in the long run).

**Fig. 6:**
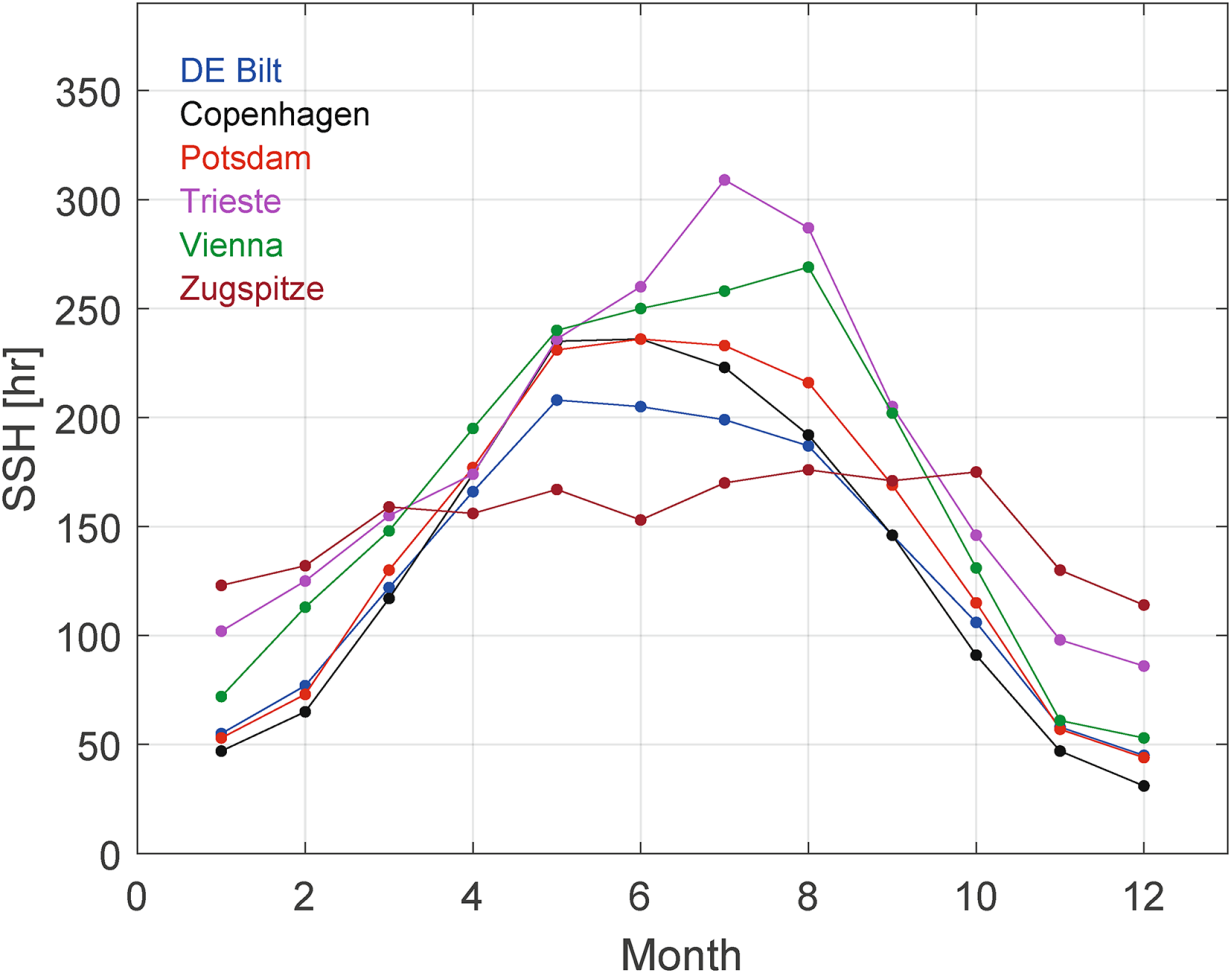
Mean monthly total SSH from Copenhagen, Potsdam, De Bilt, Vienna, Zugspitze and Trieste*.*

To complete our Fourier analysis, we applied it also to the monthly total SSH and the monthly AMO time series in order to detect specific seasonal and regional patterns. As a result, the AMO-CYC, if present at all, is mostly weaker than in the annual data and does not even appear in the Fourier spectra of each month (Figure S1, Table S1). We did not find clear trends in the monthly SSH data, except for the monthly means being different, shown in Fig. 6 (with the exception of SSH Krakow for which we had only annual data). Trieste, with its Mediterranean climate, shows the strongest seasonal variation, while Zugspitze, with its altitude of 2952 m above sea level shows the weakest.

## Discussion

The mean annual number of SSH roughly increased from north to south (Table 1); in particular, the three southernmost stations Vienna, Zugspitze and Trieste had the highest annual SSH. The reasons are complex: For example, the astronomical daylength is slightly larger in higher latitudes, but this is more than outweighed by North Atlantic low pressure systems having less influence at lower latitudes. Also, the altitude of a station influences its SSH. This is most striking in the seasonal pattern of the station Zugspitze (Fig. 6). The seasonal anomaly of the Zugspitze with its unusually high SSH in the cold season and unusually low SSH in the warm season is explained on the one hand by its height above the autumn and winter fogs and low cloud cover, and on the other hand by summer cloud formation on the mountain slopes, where warm air rises and condenses into the well-known cumulus clouds that block off the sun.

For all seven analyzed long-term annual SSH series in Central Europe, the correlation with the optimized sinusoids of the most striking period of 56–80 years (AMO-CYC), as determined by the Fourier analysis, was statistically highly significant and accounted for 18–30% of the SSH variability. The cause of the correlation is speculative to date. Different causal relations are conceivable: Firstly, the AMO, i.e. the cyclic changes in SST temperature, may influence the sunshine duration in Europe: Previous studies suggest that the AMO phases may influence the wavenumber of long waves and in the average the position of the upper ridges and upper troughs over the Eastern Atlantic, leading to changes in the frequency of anticyclonic weather^13^. However, the ultimate cause of the AMO cycles is still unclear. Alternatively, there is a common cause underlying both the SST changes in the Atlantic (AMO) and the weather in Europe (SSH): A climate modelling study in the northeast Atlantic found that the solar anomalies penetrate the ocean down to deep-water levels, leading to shifts in westerly winds^22^. Further research is needed.

The period dispersion of the AMO-CYC in the different SSH series might be caused by the link of the SSH with AMO regions of different periods, as shown in Fig. 5. This interpretation is further supported by the north-south decrease of the AMO-CYC period from the northernmost SSH Copenhagen 80 year to Potsdam 73 year, De Bilt 71 year, Krakow 64 year, Vienna 60 year, (Zugspitze 70 year), and the southernmost Trieste 56 year, which corresponds to a similar north-south decrease of the AMO periods of the Eastern North Atlantic cells (Fig. 5).

An exception is the SSH Zugspitze which does not match this trend of periods. The complex meteorological mechanism by which westerly clouds could cause the period dispersion and the observed north-south shift of SSH periods in Central Europe requires further research.

The correlation between SSH and the oscillating AMO documented here and previously^12,13^ opens up opportunities for SSH predictions. Extrapolating the AMO and SSH sinusoids into the future, we see a high likelihood of reduced sunshine in Central Europe over the next few decades. This is reliable because the existence of the AMO has been confirmed for the last 8000 years^23^, and the continuity is unlikely to change in the coming decades. Less is known about long-term SSH trends in Central Europe, but at least the high Pearson values r between the SSH and its sinusoids over more than 100 years allow a prediction of the SSH trends for the next decades. Dimming between 9 and 16% of the actual sine maximum seems likely for the seven analyzed SSH stations in the near future (Table 2, rightmost column, and Fig. 4).

The long-term trends in SSH are relevant for the solar energy production in Central Europe, both for photovoltaics and solar thermal energy. The imminent onset of a negative AMO phase with reduced SSH is likely to lead to reduced solar energy yields for at least two decades. These trends will affect the renewable energy mix of the future in Central Europe. Energy planners should consider such long-term trends to improve their projections, which are also an important basis for policy makers. The reduction in sunshine hours needs to be compensated by other renewable or non-renewable energy sources. Alternatively, the solar production capacity will need to increase even faster, coupled with large-scale battery systems. Ignoring the long-term trends in SSH associated with the AMO weakens the predictive power of future energy models and leads to unwanted surprises that can be avoided by adding empirically proven patterns from the climate history of the past > 100 years.

### Methods

#### Data

The data of seven long-term sunshine duration time series (SSH) were downloaded from the following services: De Bilt, Netherlands (1901–1922) from the climatological service KNMI^24^, Copenhagen, Denmark (1876–2020) from the DMI historical climate data collection 1768–2020 of the Danish Meteorological Institute^25^, Potsdam (1893–2022) and Zugspitze (1901–2022), both Germany, from the German Weather Service DWD^26^, Trieste, Italy (1886–2006) and Vienna, Austria (1881–2022) from HISTALP^27^. The Krakow, Poland SSH (1884–2023) was kindly provided by Dorota Matuszko^28^, author of^10^. All SSH data were monthly totals, except for the daily SSH De Bilt, which we have converted to monthly, and Krakow, which is only total annual. A few missing months, such as April 1945 from De Bilt, were interpolated. For the long-term AMO, we used the monthly data (1856–2022) from NOAA^29^ as an index of SST. For an additional analysis, we downloaded the gridded AMO data (1854–2023), monthly and unsmoothed, from NOAA^21^. From about 1880 until recently, SSH was measured with an improved Campbell-Stokes heliograph^30,31^, and afterwards with more modern sensors. In our analyses, all SSH and AMO data were left unsmoothed.

#### Fourier analysis

We applied the Fast Fourier Transform FFT with zero padding of 20,000 zeros to both the annual and monthly SSH and AMO data as anomalies - except for the monthly data for Krakow, which we did not have. Zero padding in the time domain increases the frequency resolution from 1/L to 1/(20000 + L), where L is the length of the SSH, AMO time series. This introduces more points in the same frequency range and allows interpolation between the points associated with the unpadded case^18^. However, the interpolated results of the FFT become increasingly uncertain for frequencies below 1/L (or sine periods longer than L). However, this does not affect the main results of the paper since in all cases, the AMO-CYC is considerably shorter than the L value of the SSH and AMO series.

#### Non-linear optimization

Fitting the annual SSH and AMO with optimal AMO-CYC sinusoids required two additional parameters, the phase and amplitude of the sinusoid, in addition to the known period from the Fourier analysis. We determined the phase and amplitude using the nonlinear optimization method of Nelder and Mead^19^. This method does not rely on differential quotients, uses the mean squared error MSE between the SSH and the sinusoid as the function to be minimized as the optimization criterion, and is robust to two unknowns. It is a direct search method using the concept of a simplex in n dimensions, where n is the number of unknowns, where the *n* + 1 corners of the simplex are optimally adapted in each search step.

#### Statistical processing

Our decisions of statistical significance of the Fourier Transform of the SSH, AMO are based on significance lines for the Fourier spectra. They depend on the autocorrelation of the SSH, AMO and were constructed in two steps as Detrended Fluctuation Analysis (DFA)^32–34^ and Monte Carlo simulation (MC). DFA was used to evaluate the generalized Hurst exponents α of the monthly SSH, AMO, which is a measure of the autocorrelation or long-term memory of the time series. Significance lines were then generated for reliable statistical inference on the Fourier spectral peaks using MC. The standard method of the DFA is based on monthly times series as monthly departures from the mean divided by the standard deviation. DFA with our annual time series was not feasible due to the small number of data points. For MC, we used 10,000 random synthetic data sets with the same α and length as the corresponding SSH, AMO series. The synthetic series were generated using a standard Fourier filtering procedure^35,36^. Our MC generated significance lines for the Fourier spectra with significance levels of *p* = 0.001, *p* = 0.01 and *p* = 0.05. As the Krakow data are only annual, DFA could not be applied due to the poor number of data points. To be on the safe side, we used the most unfavorable α = 0.63 of De Bilt as a guess for Krakow.

Instead of the common t-test, we preferred MC with synthetic series to calculate the significance of Pearson’s r between the SSH, AMO and the optimal sinusoids, and the significance of the linear regression lines of SSH* and AMO*, because the t-test relies on independent observations, which is not fulfilled in case of autocorrelated time series^37^. For comparison, we also tried the t-test. Only Trieste and Vienna, which are not autocorrelated, gave the same significance for the t-test and the MC. For the autocorrelated time series SSH and the AMO, the t-test generally gave lower p-values than the more reliable MC.

All annual Fourier analyses and the sinusoidal Pearson correlations r had statistical p-values of *p* < 0.001. Therefore, any correction for multiple testing such as Bonferroni would not change the results of the links between the AMO and the Central European SSH being statistically significant.

All data analysis, tables and figures in this paper were performed using Matlab version R2022b; Fig. 5, with the addition of the Matlab Mapping toolbox.

## Electronic supplementary material

Below is the link to the electronic supplementary material.

Supplementary Material 1

## Data Availability

Data is provided within the manuscript.
