# Supplementary material for: Central-European sunshine hours, relationship with the Atlantic Multidecadal Oscillation, and forecast
Source: Sci Rep. 2024 Oct 24;14:25152. doi: 10.1038/s41598-024-73506-5 (PMC11502692; doi:10.1038/s41598-024-73506-5)
Supplement: Supplementary file 1 — Supplementary Material 1 [file 41598_2024_73506_MOESM1_ESM.docx]

**Supplement to**

**Central-European Sunshine Hours, Relationship with the Atlantic Multidecadal Oscillation, and Forecast**

Horst-Joachim Lüdecke^1^, Gisela Müller-Plath^2^*, Sebastian Lüning^3^

^=================================================================^

^1^htw saar University of Applied Sciences, Germany. E-mail: [h.luedecke@htwsaar.de](mailto:h.luedecke@htwsaar.de)

^2*^Technische Universität Berlin, Berlin, Germany. E-Mail: [gisela.mueller-plath@tu-berlin.de](https://www.tu-berlin.de/allgemeine_seiten/e_mail_anfrage/id/207607/?no_cache=1&ask_mail=Xuj8gwAFbg6rt3rzxJnfsZjib6L2XmwtZ29hf3JE5MQsy5aWiG3RItfB0e%2F3CGG9&ask_name=GISELA%20MUELLER-PLATH)

^3^Institute for Hydrography, Geoecology and Climate Sciences, Hauptstraße 47, 6315 Ägeri, Switzerland. E-mail: [luening@ifhgk.org](mailto:luening@ifhgk.org)

*Corresponding author

**Contents of this file:**

[1 Fourier spectra of the monthly AMO and SSH with an AMO-CYC (peak with a significance of p < 0.05 within the period interval of ~50 to ~80 years) 2](#_Toc174197350)

[2 Table of AMO and SSH cycles between 10 and 100 years with p<0.05 10](#_Toc174197351)

[3 AMO periods of the North-Atlantic 5° N x 5° E cells 11](#_Toc174197352)

# Fourier spectra of the monthly AMO and SSH with an AMO-CYC (peak with a significance of p < 0.05 within the period interval of ~50 to ~80 years)


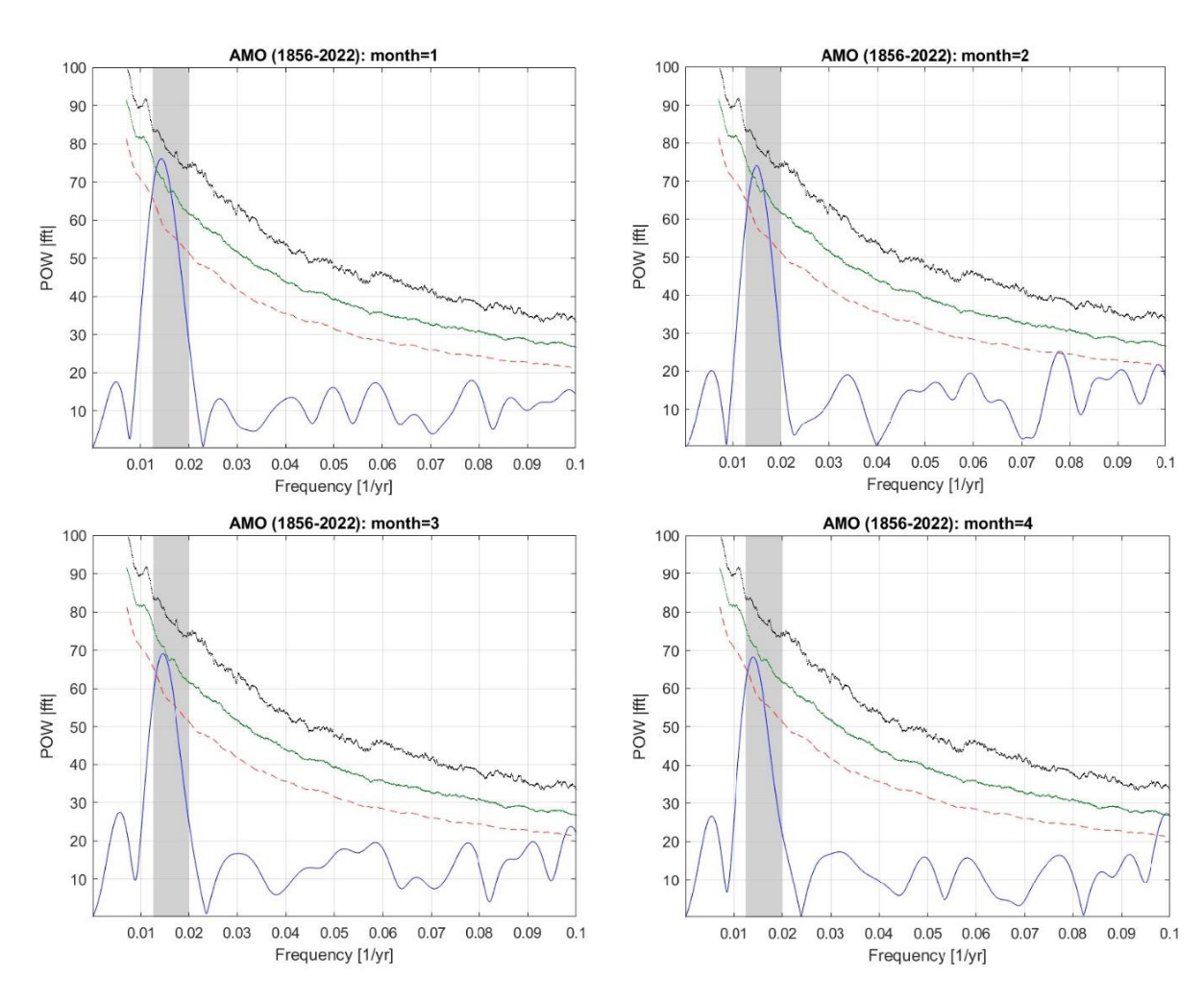


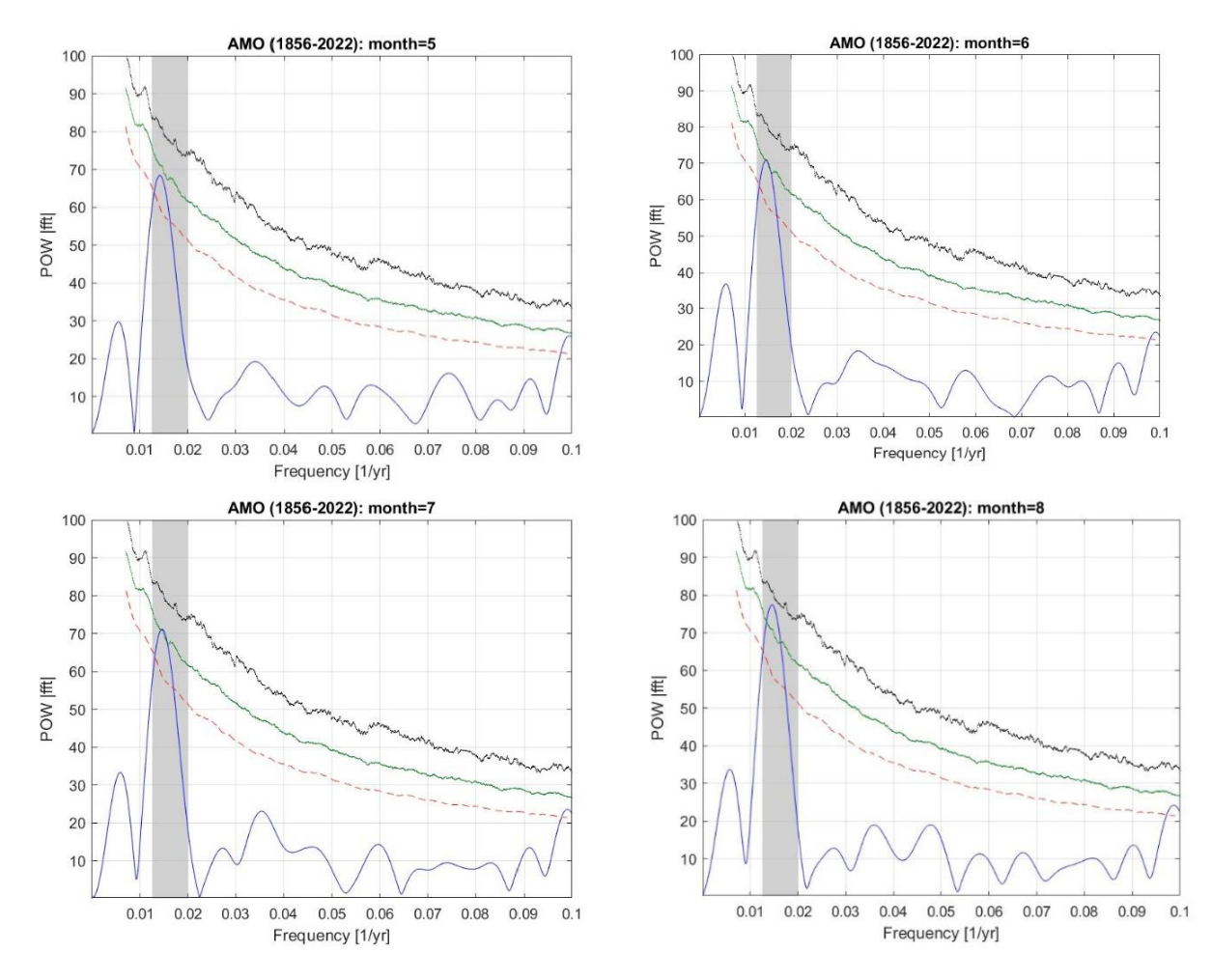


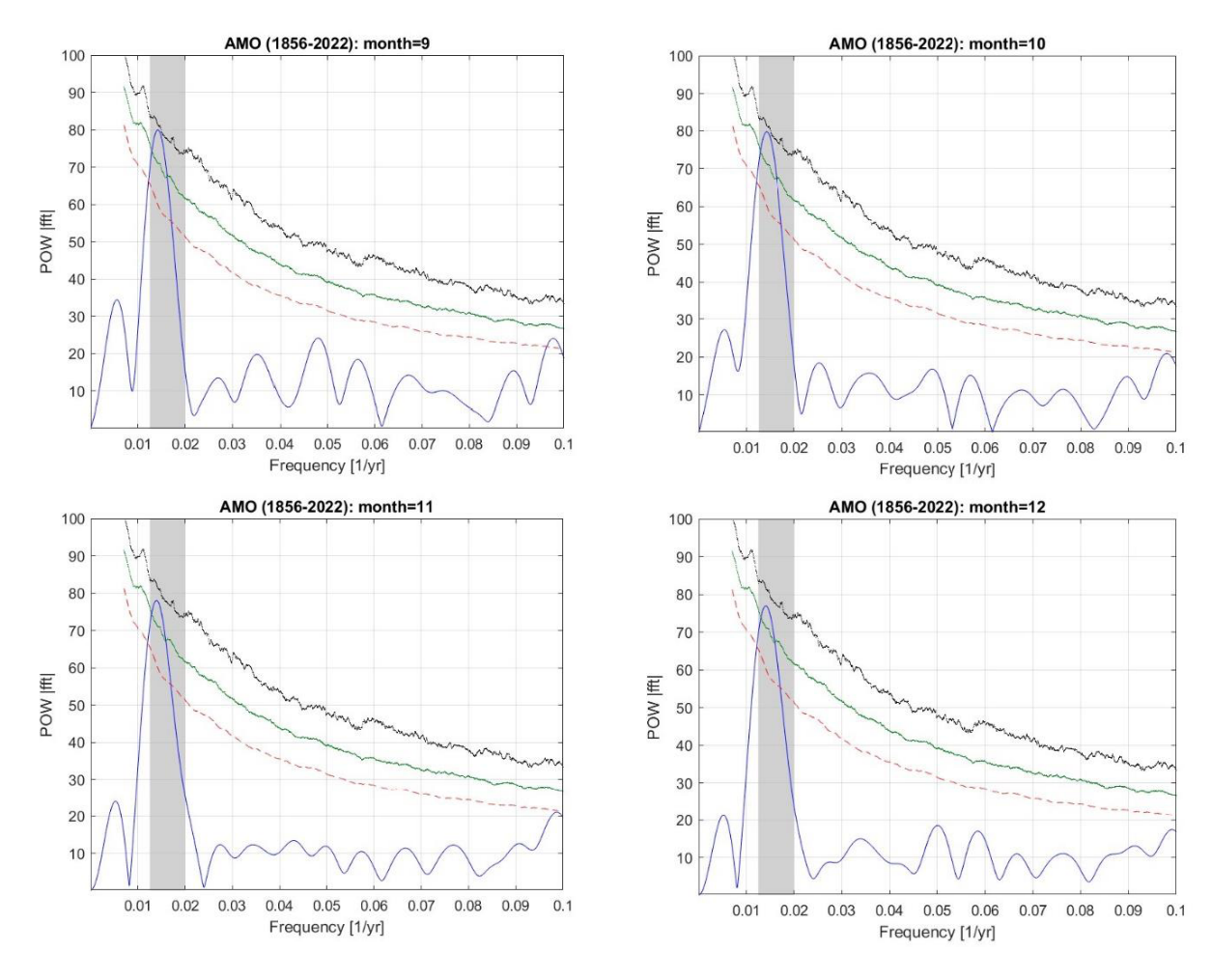


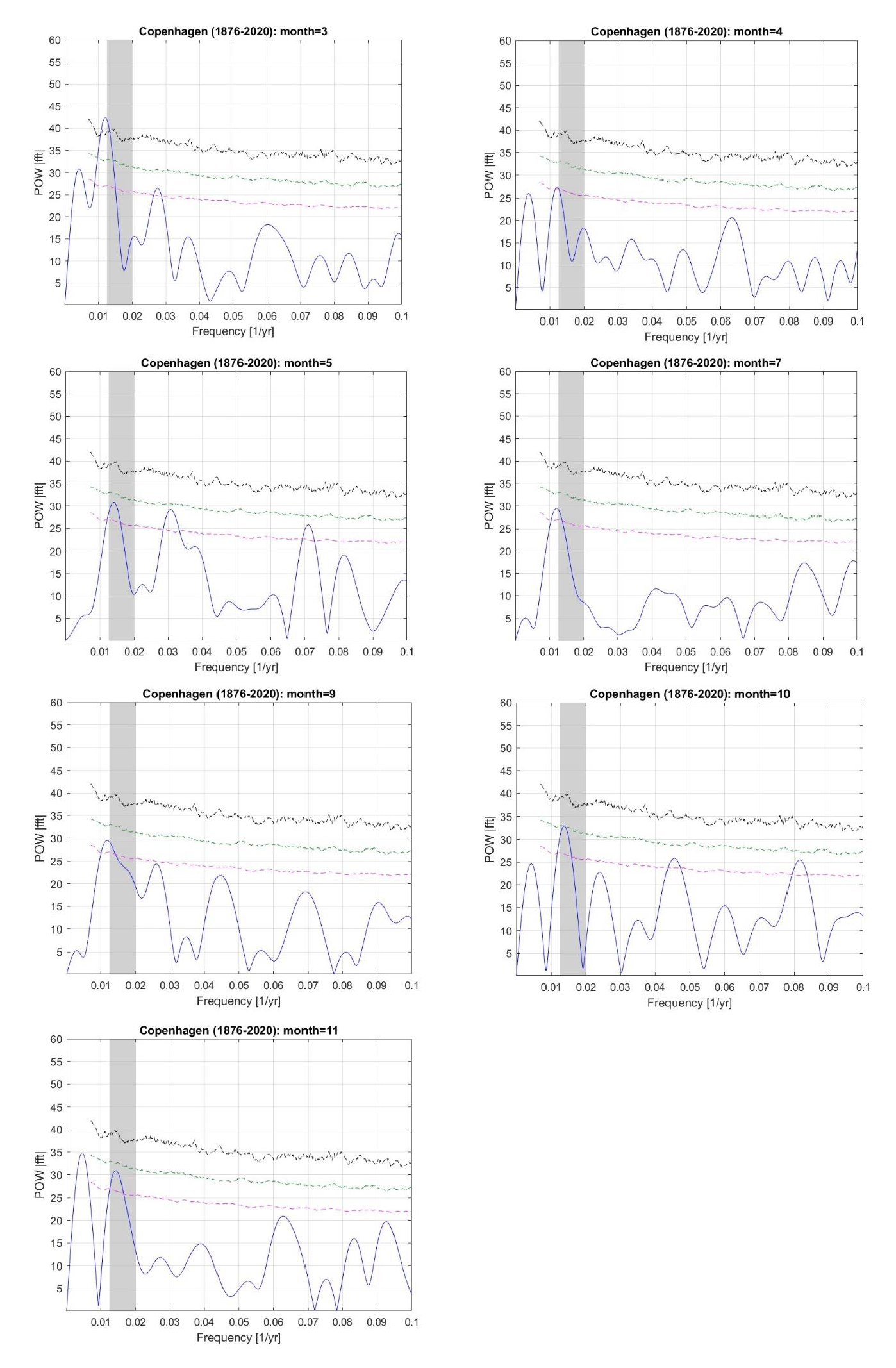


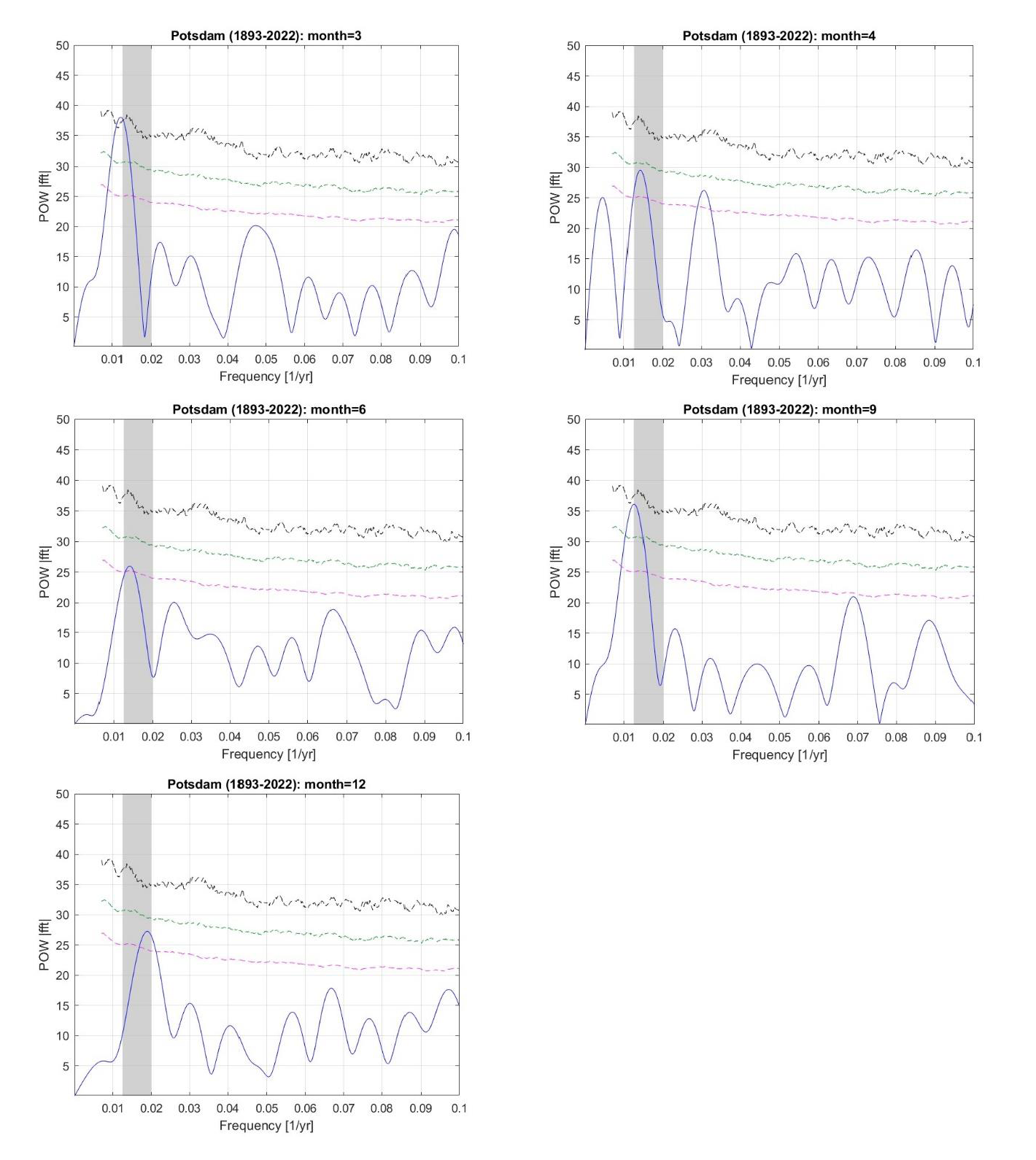


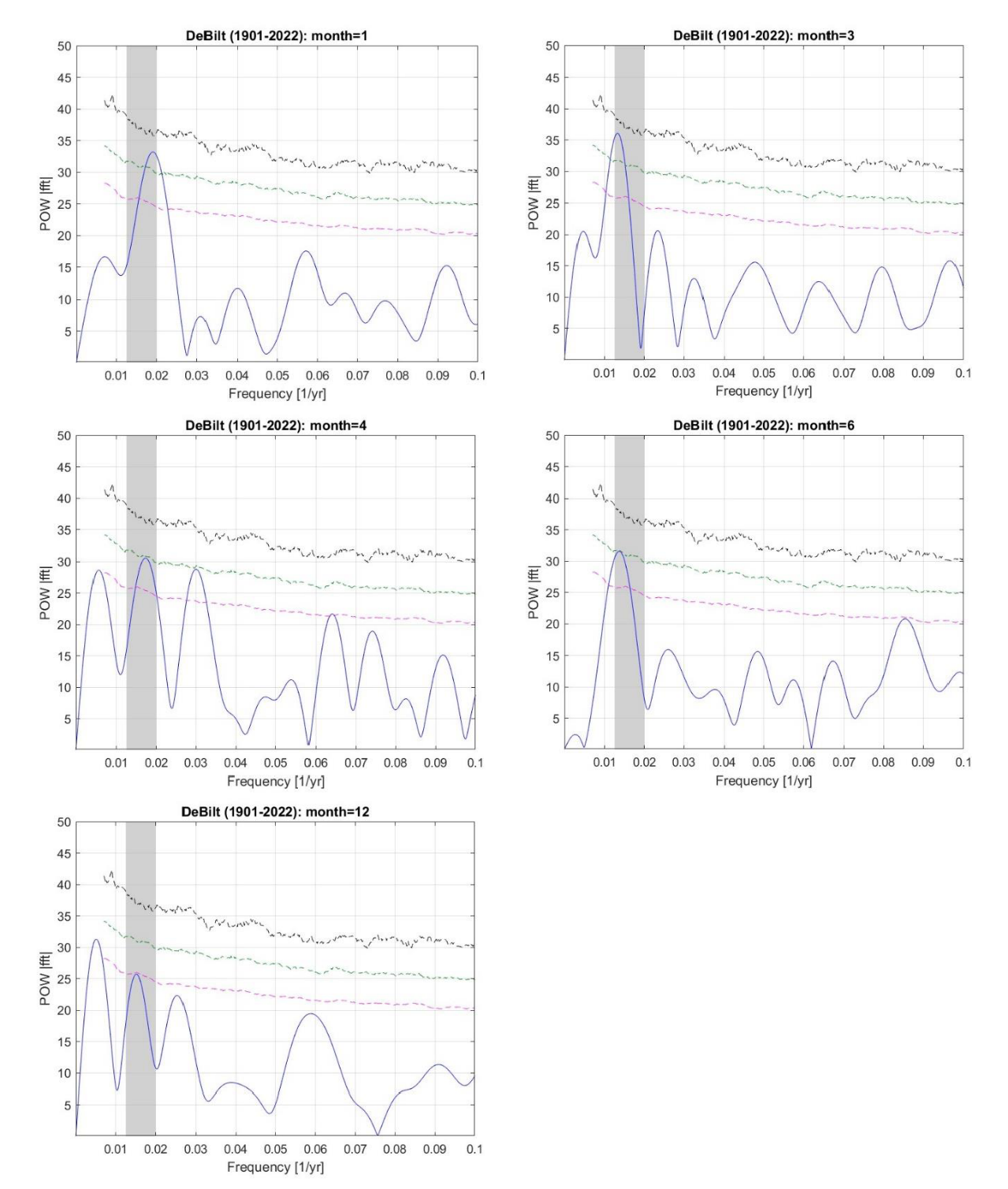


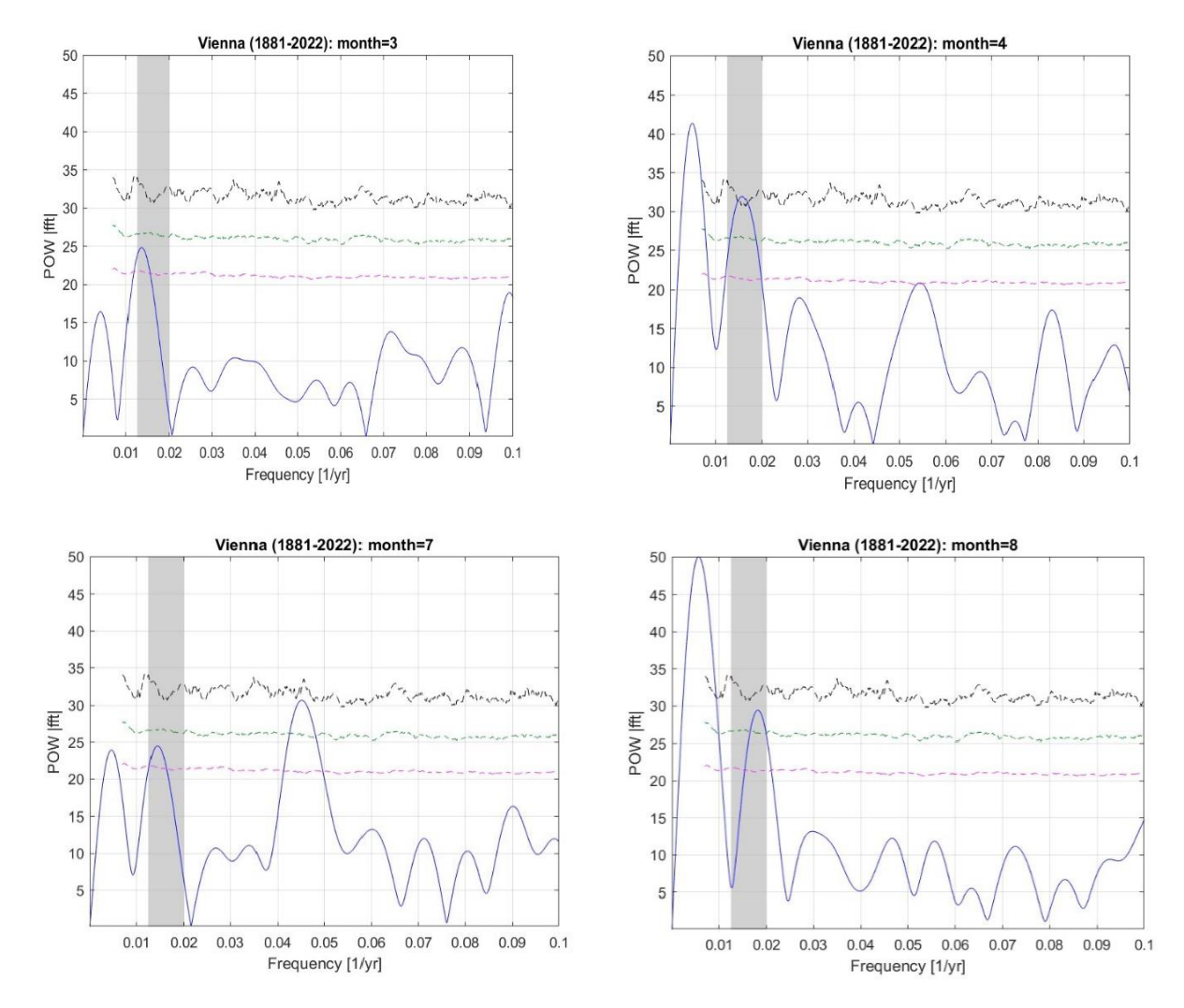


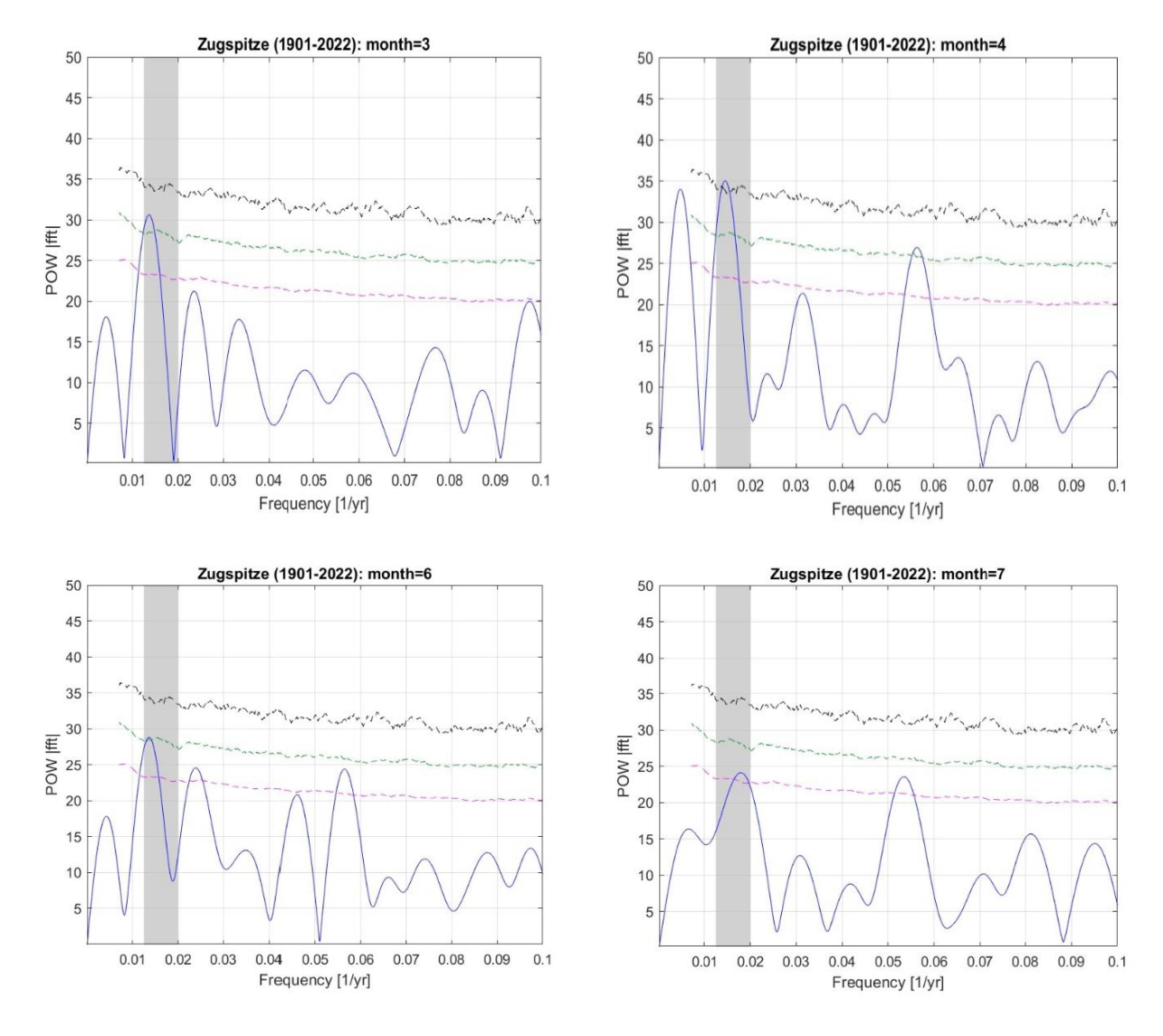


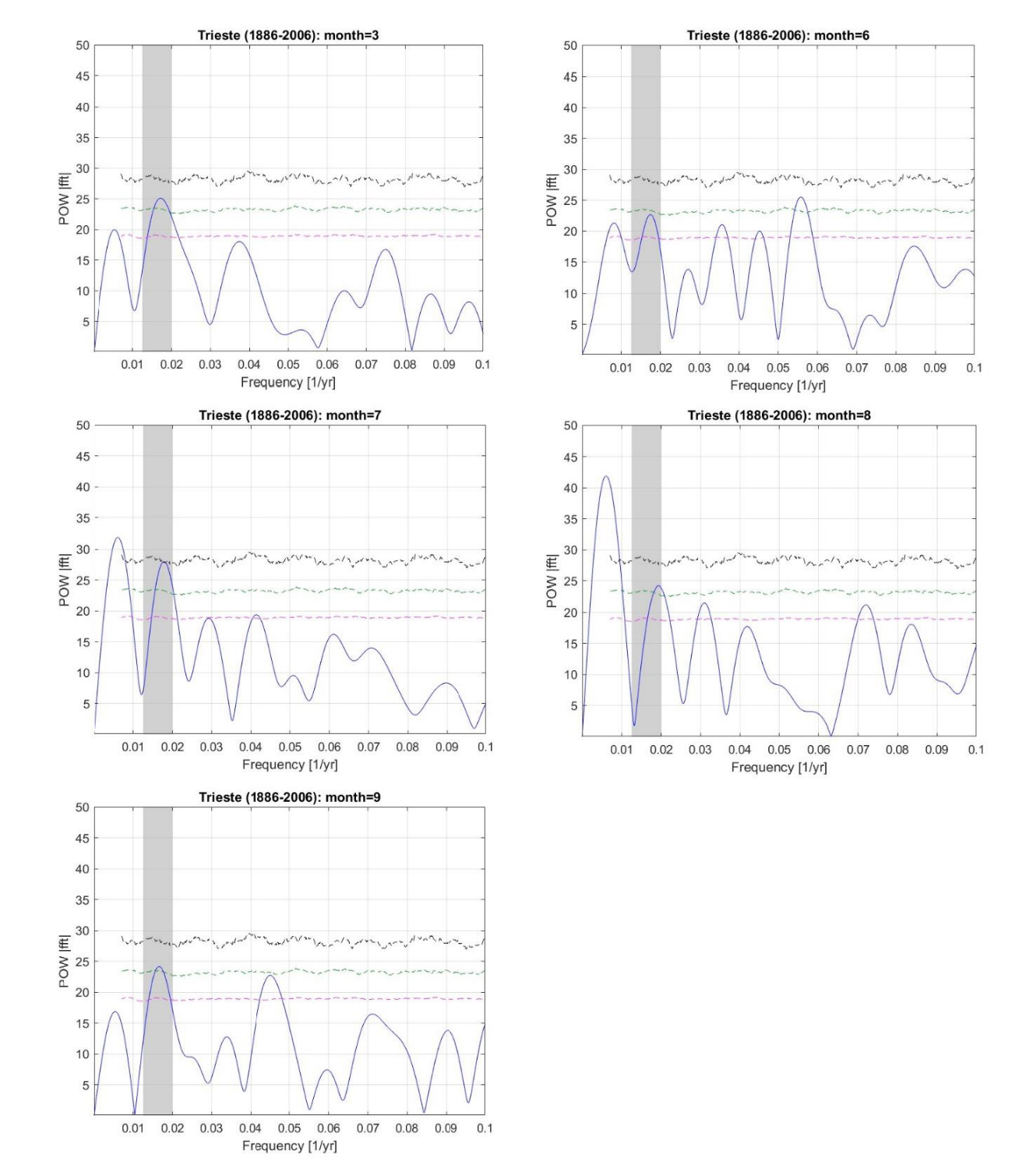


Figure S1: Some *Fourier transforms from monthly AMO and SSH from Copenhagen, Potsdam, De Bilt, Vienna, Zugspitze, and Trieste with an AMO-CYC (peak with a significance of p < 0.05 within the period interval of ~50 to ~80 years), the full list in Table S1. POW[fft]: spectral power from the Fast Fourier Transform; dashed lines: significance lines* *p = 0.001 (black), p = 0.01 (green), and p = 0.05 (red). The AMO-CYC region is indicated by the grey shaded area for clarity.*

# Table of AMO and SSH cycles between 10 and 100 years with p<0.01

| SSH, AMO | Annual |  |  |  |
| --- | --- | --- | --- | --- |
| AMO 1856-2022 | 70 |  |  |  |
| SSH Copenhagen 1876-2020 | 80 |  |  |  |
| SSH Potsdam 1893-2022 | 73 |  |  |  |
| SSH DeBilt 1901-2022 | 71 |  |  |  |
| SSH Krakow | 64 |  |  |  |
| SSH Vienna 1881-2022 | 60 |  |  |  |
| SSH Zugspitze 1901-2022 | 70 |  |  |  |
| SSH Trieste 1886-2006 | 56, 26, 14 |  |  |  |
|  |  |  |  |  |
| SSH, AMO | Jan | Feb | Mar | Apr |
| AMO 1856-2022 | 70 | 67, 13,10 | 69, 10 | 71, 10 |
| SSH Copenhagen 1876-2020 | / | / | 83, 36 | 83 |
| SSH Potsdam 1893-2022 | / | 32 | 82 | 71, 33 |
| SSH DeBilt 1901-2022 | 52 | 17 | 76 | 58, 33, 16 |
| SSH Vienna 1881-2022 | 25, 20, 16 | 36 | 74 | 64 |
| SSH Zugspitze 1901-2022 | / | / | 73 | 69, 18 |
| SSH Trieste 1886-2006 | 19 | / | 58 | 17 |

| SSH, AMO | May | Jun | | Jul | Aug |
| --- | --- | --- | --- | --- | --- |
| AMO 1856-2022 | 70, 10 | 69, 10 | | 69, 10 | 69, 10 |
| SSH Copenhagen 1876-2020 | 72, 32, 14 | / | | 84 | 25 |
| SSH Potsdam 1893-2022 | 10 | 71 | | 12 | / |
| SSH DeBilt 1901-2022 | 10 | 73 | | / | 22 |
| SSH Vienna 1881-2022 | 28 | / | | 70, 22 | 55 |
| SSH Zugspitze 1901-2022 | / | 73, 42, 18 | | 56, 19 | / |
| SSH Trieste 1886-2006 | 40, 28 | 57, 28, 22, 18 | | 56, 24 | 52, 32, 14 |
|  |  |  |  | |  |
| SSH, AMO | Sep | Oct | Nov | | Dec |
| AMO 1856-2022 | 71, 10 | 71 | 72 | | 71 |
| SSH Copenhagen 1876-2020 | 84 | 73, 22, 12 | 70 | | 33 |
| SSH Potsdam 1893-2022 | 79 | 20, 10 | / | | 53 |
| SSH DeBilt 1901-2022 | / | 14, 11 | / | | 66 |
| SSH Vienna 1881-2022 | / | / | / | | 15 |
| SSH Zugspitze 1901-2022 | / | / | / | | 11 |
| SSH Trieste 1886-2006 | 60, 22 | / | / | | 23 |

Table S1: *Periods of the Fourier-transform annual and monthly for January until December between 10 to 90 yrs of SSH and the AMO with significance p < 0.05 (red), p < 0.01 (green) and p < 0.001 (black). Periods longer than 90 years are unreliable in the Fourier transform of the length of our SSH series and are not shown here.*

# AMO periods of the North-Atlantic 5° N x 5° E cells

| lon lat per [yr]  2.5 -42.5 60  2.5 -37.5 60  2.5 -47.5 61  2.5 -32.5 61  7.5 -52.5 61  7.5 -32.5 61  12.5 -62.5 61  12.5 -57.5 61  7.5 -47.5 62  7.5 -42.5 62  7.5 -37.5 62  12.5 -52.5 62  12.5 -47.5 62  17.5 -62.5 62  17.5 -57.5 62  62.5 -2.5 62  2.5 -27.5 63  7.5 -27.5 63  12.5 -42.5 63  12.5 -37.5 63  12.5 -32.5 63  17.5 -52.5 64  17.5 -47.5 64  17.5 -42.5 64  17.5 -37.5 64  17.5 -32.5 64  17.5 -27.5 64  62.5 -57.5 64  62.5 -52.5 64  62.5 -7.5 64  7.5 -22.5 65  12.5 -27.5 65  22.5 -32.5 65  22.5 -27.5 65  22.5 -22.5 65  57.5 -57.5 65  57.5 -52.5 65  57.5 -47.5 65  -7.5 2.5 66  17.5 -22.5 66  22.5 -47.5 66  62.5 -12.5 66  57.5 -7.5 66  -7.5 -12.5 67  -7.5 -7.5 67  -7.5 -2.5 67  -2.5 -32.5 67  7.5 -17.5 67  12.5 -22.5 67  22.5 -62.5 67  22.5 -57.5 67  22.5 -52.5 67  22.5 -42.5 67  22.5 -37.5 67  27.5 -22.5 67  27.5 -17.5 67  7.5 -12.5 68  27.5 -27.5 68 | lon lat per [yr]  32.5 -17.5 68  -2.5 -7.5 69  32.5 -22.5 69  52.5 -47.5 69  57.5 -42.5 69  57.5 -12.5 69  62.5 -37.5 69  62.5 -22.5 69  -7.5 -27.5 70  -7.5 -22.5 70  -7.5 -17.5 70  -2.5 -12.5 70  2.5 -12.5 70  2.5 -7.5 70  2.5 -2.5 70  22.5 -67.5 70  32.5 -12.5 70  52.5 -52.5 70  52.5 -42.5 70  57.5 -37.5 70  57.5 -32.5 70  57.5 -27.5 70  57.5 -22.5 70  57.5 -17.5 70  62.5 -27.5 70  62.5 -17.5 70  -2.5 -27.5 71  -2.5 -2.5 71  22.5 -72.5 71  27.5 -67.5 71  27.5 -62.5 71  27.5 -57.5 71  37.5 -27.5 71  37.5 -22.5 71  37.5 -17.5 71  37.5 -12.5 71  42.5 -32.5 71  42.5 -27.5 71  42.5 -22.5 71  42.5 -17.5 71  42.5 -12.5 71  47.5 -47.5 71  47.5 -32.5 71  47.5 -27.5 71  52.5 -37.5 71  52.5 -32.5 71  52.5 -22.5 71  52.5 -17.5 71  52.5 -12.5 71  -7.5 -32.5 72  2.5 2.5 72  27.5 -52.5 72  32.5 -57.5 72  32.5 -52.5 72  37.5 -32.5 72  47.5 -52.5 72  47.5 -22.5 72  47.5 -17.5 72 | lon lat per [yr]  47.5 -12.5 72  47.5 -7.5 72  52.5 -27.5 72  62.5 -32.5 72  27.5 -72.5 73  27.5 -47.5 73  27.5 -32.5 73  32.5 -67.5 73  32.5 -62.5 73  32.5 -47.5 73  32.5 -27.5 73  37.5 -52.5 73  37.5 -47.5 73  37.5 -42.5 73  37.5 -37.5 73  42.5 -37.5 73  47.5 -42.5 73  47.5 -37.5 73  -2.5 -22.5 74  -2.5 -17.5 74  42.5 -47.5 74  42.5 -42.5 74  2.5 -17.5 75  27.5 -42.5 75  32.5 -72.5 75  32.5 -42.5 75  37.5 -57.5 75  42.5 -67.5 75  42.5 -52.5 75  2.5 7.5 76  27.5 -37.5 76  32.5 -37.5 76  32.5 -32.5 76  37.5 -72.5 76  37.5 -67.5 76  27.5 -77.5 77  37.5 -62.5 77  42.5 -62.5 77  42.5 -57.5 77  2.5 -22.5 78  -2.5 2.5 79  32.5 -77.5 81  -7.5 7.5 82  -2.5 7.5 83  67.5 -17.5 83  57.5 2.5 83  72.5 -17.5 85  72.5 7.5 85  72.5 -12.5 86  62.5 2.5 87  67.5 7.5 87  72.5 -7.5 87  72.5 2.5 87  72.5 -2.5 88  67.5 2.5 89  67.5 -12.5 90  67.5 -7.5 91  67.5 -2.5 92 |
| --- | --- | --- |

Table S2: *Geographical distribution of AMO cycle periods in the 5° N x 5° E grid cells of the Atlantic region (10° S - 80° N, -10° W - 80° W). lon, lat: longitude, latitude of the grid cell center; per: period [yr] computed with FFT.*
